# A Study Protocol for a Randomized, Controlled Trial: Improving Glucose Time-in-Range in Diabetes in African Youth (DAYTime)

**DOI:** 10.3390/mps9020043

**Published:** 2026-03-08

**Authors:** Thereza Piloya-Were, Catherine Nyangabyaki, Elizabeth Pappenfus, Expeditus Ahimbisibwe, Ezrah Trevor Rwakinanga, Lin Zhang, Silver Bahendeka, Antoinette Moran

**Affiliations:** 1 Department of Paediatrics, School of Medicine, College of Health Sciences, Makerere University, Kampala 256, Uganda; 2 St. Francis Hospital Nsambya, Kampala 256, Uganda; cadyerin@gmail.com (C.N.);; 3 Department of Pediatrics, University of Minnesota, Minneapolis, MN 55454, USA; 4 Ministry of Health, Kampala 256, Uganda; 5 Division of Biostatistics, Department of Health, University of Minnesota, Minneapolis, MN 55414, USA

**Keywords:** type 1 diabetes, continuous glucose monitoring, less-resourced nations, adolescents, children

## Abstract

Metabolic control is poor in East Africa for youth with type1 diabetes (T1D). Self-monitoring of blood glucose (SMBG) by fingerstick 2–3 times daily is routine care. This randomized controlled trial (RCT) will test the hypothesis that providing continuous glucose monitoring (CGM) to Ugandan youth with T1D will improve glucose time-in-range (TIR glucose 3.9–10.0 mmol/L) and be cost effective in this setting. Ugandan youth with T1D (*n* = 180, age 4–26 years) will be divided into four 12-month cohorts (August 2022–August 2027). Half will receive unblinded Freestyle Libre 2 Flash CGM for 12 months. For six months, control subjects received sufficient test strips for SMBG three times daily while wearing blinded Freestyle Libre Pro CGM (for endpoint assessment), and then they switch to unblinded CGM for six months. Everyone receives monthly diabetes education. The primary endpoints are as follows: (1) the six-month change from baseline in glucose TIR, unblinded CGM versus SMBG; (2) a cost analysis of CGM versus SMBG. The TIR hypothesis will be tested by linear mixed effects models. Cost analysis assumptions include direct material and indirect costs like hospitalizations, missed school/work, and diabetes complications. The study will inform T1D management guidelines in a low resource setting using evidence-based recommendations.

## 1. Introduction

Although the data are incomplete due to inconsistent reporting, the International Diabetes Federation (IDF) reported that in 2024 about 352,000 individuals with T1D were living in Africa, ~98,000 of whom (28%) were children [1]. The prevalence continues to rise as detection, reporting, and survival improve [1]. In Uganda, ~6000 children and young [1] adults now live with T1D [1].

Ugandan youth up to age 26 are followed monthly in dedicated diabetes clinics staffed by trained personnel, and receive adequate quantities of insulin, diabetes education, and 2–3 glucose test strips per day. This level of care is considered to be within the diabetes “intermediate care” tier [2]. Metabolic control is poor in this population, with extremes of both hypo- and hyperglycemia and average hemoglobin A1c (HbA1c) levels of ~11% (97 mmol/mol) [3]. Both acute and chronic life-threatening diabetes complications are common. Thus, the current level of T1D management is inadequate.

Glucose monitoring is an essential component of diabetes care, and more frequent monitoring is associated with lower HbA1c levels [4]. International T1D guidelines recommend SMBG at least 6–10 times per day [5,6]. GM is rapidly replacing SMBG as standard-of-care in high-resourced nations. It is associated with lower HbA1c levels, increased glucose TIR, and less hypoglycemia [7,8,9,10,11,12]. The American Diabetes Association recommends access to CGM from the outset of T1D diagnosis [6].

CGM is currently considered too expensive for low-resource settings. However, frequent glucose monitoring by any means is expensive, and CGM devices have been shown in high income countries to be cost effective compared to SMBG. A systematic review on the cost effectiveness of CGM in people with TID suggests CGM appears to be a cost-effective tool by reducing short- and long-term complications for individuals with type 1 diabetes [13]. Another study in Sweden showed benefit of Flash CGM over SMBG in hypoglycemia and health utility benefits, which may ultimately lead to economic benefit [14]. A 2020 US cost comparison found that flash glucose monitoring was equivalent in price to three times daily SMBG [15]. In addition to direct material costs, cost-benefit calculations consider avoidance of diabetic ketoacidosis (DKA) and severe hypoglycemia, preventing micro- and macrovascular complications, providing the opportunity to achieve normal life milestones (education, sports participation, employment, marriage, having children) and being able to expect normal longevity.

This protocol aims to determine whether CGM leads to significant improvement in diabetes metabolic control in Ugandan youth with T1D, and includes cost-benefit analysis by the Ugandan Ministry of Health since the economic realities of this technology may differ between low- and high-income settings. These data will help inform treatment decisions in Uganda and may be applicable to other low-resource countries. The study also provides an opportunity for the experienced Minnesota research team to train and mentor the Ugandan pediatric diabetes team in the conduct of RCTs, thus increasing local research capacity. The protocol follows the Standard Protocol Items Recommendations for Interventional Trials (SPIRIT) details in Table S1.

## 2. Methods

### 2.1. Study Methods Development

This study grew out of a long-standing clinical partnership between Dr. Antoinette Moran at the University of Minnesota and Dr. Theresa Piloya-Were at Mulago Hospital, the Ugandan national referral hospital and the teaching hospital of Makerere University. In preparation, they performed a pilot study of 78 East African youths using a single, blinded CGM wear, to assess feasibility and participant acceptance of this technology and to generate data for sample size estimation [3]. While planning the current RCT, they considered several challenges and potential limitations that informed the ultimate approach, as outlined in Table A1. Goals important to the investigators included ensuring there was a true control group receiving standard SMBG care (with simultaneous blinded sensor wear for endpoint assessment), allowing every participant to experience unblinded CGM use (the control group crosses over to unblinded CGM after the first six months), and assessing the durability of CGM benefits beyond the six-month primary endpoint (the CGM group continues until 12 months).

### 2.2. Objectives

The primary objectives of this study are outlined as follows:

1. To determine if patients’ ability to continuously observe interstitial glucose levels for six months using the Freestyle Libre 2 flash CGM device (Abbott Diabetes Care, Alameda, CA, USA) improves glucose TIR from baseline assessment. The change in glucose TIR while wearing the unblinded CGM will be compared to change in TIR in patients performing three times daily SMBG.
2. To perform a cost analysis on flash glucose monitoring compared to three times daily SMBG, to determine whether this technology is cost effective in the setting of a less-resourced nation.

Secondary objectives are as follows: to assess the change-from-baseline impact of unblinded CGM on percent TIR at 12 months; percent time with glucose in hyper- and hypoglycemic ranges; glucose variability (coefficient of variation, CV); HbA1c; patient satisfaction, quality of life, and fear of hypoglycemia. There are also training objectives for the Ugandan research team and the Kampala diabetes community.

### 2.3. Overview, Study Design

This randomized, non-blinded, phase 4 clinical trial is programmed as follows. Subjects are assigned 1:1 to the CGM Group or the Control Group. Randomization is stratified by clinic and by age group (4–11, 12–18, and 19–26 years) with approximately equal numbers in each age group and of the two clinic locations. The random numbers were generated by the statistician using a central computer. At baseline, a 1–2 weeks initial assessment is performed where the ability to wear and return the sensor can be demonstrated, as an entry criterion for randomization. After this, 180 children and young adults with T1D will be randomized into the clinical trial in four 12-month cohorts of 45 patients each.

**Figure 1:**
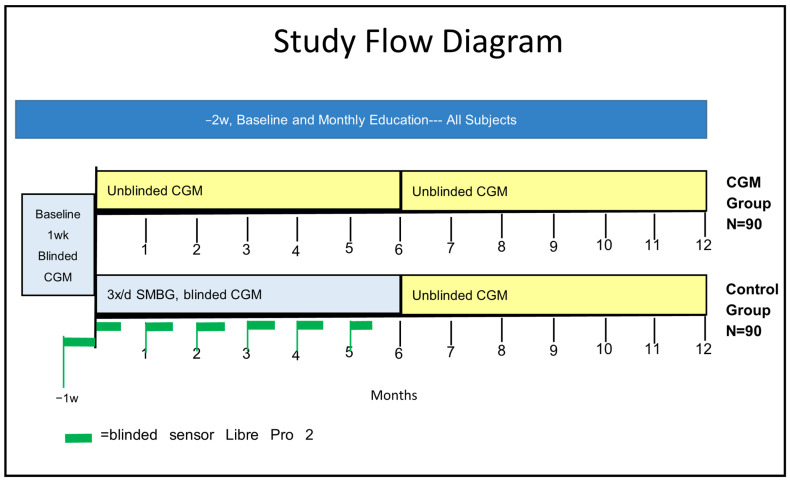
Study flow diagram figure legend. Dark green color is librepro wear for 2wks, yellow is unblinded CGM wear, blue is for Blinded CGM wear.

The study design is shown in Figure 1. Half of the subjects (*n* = 90, CGM Group) receive unblinded FreeStyle Libre 2 CGM for the entire 12 months. Half (*n* = 90, Control Group) are given sufficient test strips for three times daily SMBG months 1–6, while wearing blinded CGM for endpoint assessment. For months 7–12 they switch to unblinded CGM. The treatment group is expected to continuously modify their insulin dose and behaviors based on real-time, continuous availability of glucose data. The control group has no access to these data and will base their actions on SMBG measurement alone. For study data analysis, we will use unblinded sensor data from the treatment group and blinded sensor data from the control group that is obtained in the two weeks preceding each monthly visit; thus, the CGM data comparison period will be the same for both groups.

The investigators and the patients are not blinded to treatment arm as there is no practical way to do this. But they are blinded to the comparison data for CGM versus SMBG use until the study is completed. Table 1 lists the schedule of study assessments.

A baseline 1–2 weeks initial assessment will be performed where the ability to wear and return the CGM can be demonstrated as an entry criterion for study randomization. After this, 180 children and young adults with T1D will be randomized to a 12-month clinical trial stratified by age and clinics (Mulago or Nsambya) to determine if the availability to see continuous glucose levels by Freestyle Libre 2 CGM will improve glucose TIR compared to SMBG 3x/day. All subjects will receive intensive monthly diabetes self-management education. Half of the subjects (*n* = 90, “CGM Group”) will be given unblinded FreeStyle Libre 2 CGM for the entire 12 months. Half of the subjects (*n* = 90, “Control Group”) will be given sufficient test strips for 3x daily SMBG levels months 1–6. They will wear blinded CGM for endpoint assessment. For months 7–12 they will switch to unblinded CGM.

Primary endpoint assessment occurs after six months of unblinded CGM (months 1–6 for the CGM Group, and 6–12 for the Control Group). The first six months of the Control Group will serve as the control. The CGM Group will receive an additional six months (months 7–12) of unblinded CGM to determine the effects of longer use of CGM wear.

### 2.4. Study Setting, Recruitment and Consent

This study is being conducted in two urban hospital-based T1D clinics in Kampala, Uganda. Mulago Hospital (“Mulago”) is the national referral hospital and the teaching hospital for Makerere University. Nearby, St. Francis Hospital, Nsambya (“Nsambya”) is a teaching hospital for Mother Kevin Post Graduate Medical School, Uganda Martyrs University. These are the two largest pediatric diabetes clinics in Uganda. Patients are recruited through their pediatric diabetes clinics. At Mulago, T1D care is supported by a pediatric endocrinologist, residents, 2 nurses and a dietitian. At Nsambya, the diabetes team is headed by a pediatrician and is supported by 2–3 nurses plus residents. Each clinic follows about 300 children and young adults age 0–26 years. Nsambya is a private not-for-profit missionary hospital while Mulago is a public government hospital. The patients seen at Nsambya have a moderately higher socioeconomic status than those seen at Mulago, but only a few are able to afford better health care or insurance. Both clinics are supported by the CDiC^®^ program (Changing Diabetes in Children, Novo Nordisk, Denmark), with patients receiving free insulin, 2–3 glucose strips per day for SMBG, measurement of HbA1c levels every three months, diabetes education, and an occasional diabetes camp for psychosocial support. Subjects are recruited by the local diabetes teams from their clinics. Patients are commonly seen in pediatric diabetes clinics up to age 26.

### 2.5. Eligibility Criteria

Eligibility criteria include living in the Kampala, Uganda area, age 4–26 years, T1D (determined by clinical criteria) of at least 12 months duration, receiving insulin therapy, and having access to a cell phone (nearly ubiquitous in Uganda).

Exclusion criteria include those unwilling or unable to be seen monthly at the clinic, pregnant or breast-feeding or likely to become pregnant in the next year, any condition which the investigator feels would interfere with study participation, that the patient already has CGM (very rare in Uganda), and inability to wear the sensor for at least 7 days or return it during the baseline assessment period.

### 2.6. Study Management

Both study groups receive intensive monthly diabetes self-management education, with a focus on pattern recognition and insulin adjustment to prevent hypo- and hyperglycemia. Between visits, they have unlimited availability to contact study personnel by telephone at all times. Participants return used sensors at each visit. All study procedures are performed by trained personnel. CGM is considered a low-risk medical intervention.

Participants wearing the unblinded FreeStyle Libre 2 CGM system are able to see their glucose levels at all times. The research team is able to use these data for insulin adjustment, and participants are taught how to interpret the data. Subjects in the control arm wear the blinded CGM Libre Pro while performing SMBG three times daily for six months. Patients are taught how to interpret SMBG data and the research team uses these data for insulin adjustment. Blinded FreeStyle Libre Pro CGM sensors, placed monthly, are used to provide control data. The study team uploads the de-identified data to a study website and neither participants nor the research teams have access to the blinded sensor data for clinical use until the end of months 0–6, to preserve the blinding.

Participants are prescribed insulin regimens as per the patient’s usual routine. This was generally human NPH and regular insulin. However, analog insulin, longacting Basaglar and Levemir and rapid novorapid are increasingly becoming widely available. No participants in the study will be on premixed insulin. The insulin pens are stored at room temperature for the majority of the participants.

### 2.7. Sample Size Determination

The sample size is determined based on hypothesis testing of the primary efficacy endpoint. The null hypothesis is that the two arms will demonstrate no significant differences in the six-month change in glucose TIR. The alternative hypothesis is that wearing unblinded CGM will have greater improvement in glucose TIR over six months compared to the control arm. We estimate a standard deviation of 16.6% for baseline TIR based on our pilot data [3] and assume a moderate correlation of 0.5 between baseline and 6-month TIR values. A sample size of 144 patients (72 per arm) will have over 90% power to detect a difference of 8.6% in 6-month TIR changes between the two arms at the one-sided significance level of 0.05. To allow a 20% dropout rate, a total of 180 patients will be randomized with planned study completion of at least 144 patients. The sample size will have over 90% power to detect a 5-percentage point difference in the 6-month change from baseline in percent time < 70 mg/dL (3.9 mmol/L) or a 3.5 percentage point difference in percent time < 54 mg/dL (3.0 mmol/L) at the one-sided significance level of 0.05.

### 2.8. Statistical Methods

#### 2.8.1. Primary Efficacy Endpoint

The primary efficacy endpoint is the six-month change in percent TIR. A mixed effects model will be used, with a subject-level random intercept, with the fixed-effect predictors including the time of measurement (0, 6, or 12 month), time-dependent binary indicator for the type of CGM (SMBG with blinded CGM or unblinded CGM), and time-by-CGM-type interaction. Covariates such as age and gender will also included if tested as significant in univariate analysis.

#### 2.8.2. Cost Analyses

Primary cost analysis: material cost of the Libre CGM system vs. SMBG

We calculated the cost of three times daily SMBG per patient per 1 year. Assumptions to consider included provision of one glucometer per patient per year plus a replacement in up to 20% of patients in the second half of the year, batteries for the glucometer every 6 months, 3 test strips per day per patient plus 10% for supplementing those who might need additional support (such as during acute illness), 1 lancet device per patient per year with replacement of up to 50% in the second half of the year, and two packets of 200 lancets per patient per year. We also calculated the cost of SMBG six times daily per patient per 1 year, since international consensus standards state that SMBG should occur 6–10 times per day for optimum care.

We calculated the direct cost of the Freestyle Libre 2 CGM system per patient per 1 year. This model includes provision of 1 Freestyle Libre 2 CGM reader per patient including rechargeable batteries, 26 sensors per year (one every 14 days), glucose test strips for confirmation of readings four times per week (the CGM system has glucose monitoring capacity, so no separate meter is necessary), one lancet device, and 50 lancets. We assume 10% extra of these supplies for replacement or increased needs, except for sensors, where we assume 20% extra are needed.

#### 2.8.3. Secondary Cost Analyses

For each patient in each group per year, we will calculate the cost of hospital admissions for severe hypoglycemia and DKA as well as days missed from school or work. We will also estimate future long-term costs to the healthcare system. Diabetes chronic complications occur after many years of poor diabetes control and are not likely to appear during this study, although they could develop earlier in African youth with T1D because of chronic poor control. We will calculate the average health care cost in Uganda for retinopathy, renal failure, foot ulcer care, and amputation. There are good data available documenting the specific risk reductions associated with specific improvements in glucose TIR, and we will use these in our calculations. The hazard rate of development of retinopathy progression is reduced by 64% and development of microalbuminuria is reduced by 40% for each 10-point increase in glucose time-in-range [16]. Based on the literature, we expecting to achieve at least a 10-point improvement in glucose time-in-range in patients using CGM [17] and at the end of the study we will use our actual rates of improvement to calculate anticipated future risk reduction for retinopathy and microalbuminuria using published data [16].

In a study in Northern Uganda looking at disability from limb loss [18], the economic burden of being disabled was twofold—inability to work and the extra costs associated with accessing disability services in distant places. This will be a dimension which we will explore and try to cost each aspect as it affects individual patients and their households.

Summary statistics will be calculated for each cost endpoint. The primary and secondary endpoints will be compared between the CGM and SMBG groups using 2-sample *t*-tests or the Wilcoxon rank-sum tests. Linear regression models will also be used for the group comparisons with adjustment for important covariates such as age, gender, and education levels.

#### 2.8.4. Additional Analyses

Analysis of demographics and baseline characteristics will be descriptive with appropriate summary statistics calculated. Subgroup analyses for the primary endpoints for sensitivity analyses will be conducted. All primary analyses will follow the intent-to-treat principle. The maximum likelihood estimation method will be used to deal with any missing data, assuming that data are missing at random. Sensitivity analyses will be carried out with various assumptions of missing mechanisms. Unblinded CGM offers the prospect of direct benefit with the potential to reduce hyper- and hypoglycemia in the concerned population and has been shown to be safe and effective in children, and we want to collect data for other variables such as DKA and serious hypoglycemia even if the unblinded CGM has no efficacy on TIR; thus, no interim analysis is planned.

### 2.9. Study Administration

The UMN Coordinating Center is led by Dr. Antoinette Moran, who has overall responsibility for the study. The Coordinating Center is responsible for maintaining quality assurance and quality control systems to ensure that the trial is conducted and data generated, documented, and reported in compliance with the protocol, Good Clinical Practice and applicable regulatory requirements. Dr. Thereza Piloya-Were, the Ugandan PI, has responsibility for all study activities in that country. Mr. Expeditus Ahimbisibwe and Mr. Ezrah Trevor Rwakinanga from the Ugandan Ministry of Health (MOH) is performing the cost analyses and is responsible for presenting these findings to the MOH to assist in policy determination. A DSMB with two members from East Africa and one from the US met by conference call every 6 months. As this is a low-risk study, there is no external monitor, but the internal monitoring is done by the DSMB. Any protocol modifications are reported to both the local and Minnesota Research Ethic Committees. All records at UMN and in Uganda are kept in a secure location and will be kept for as long a period as dictated by local IRB and Institutional regulations.

If any serious unanticipated adverse device effect (UADE) occurs, the use of the study device may be suspended or stopped completely, pending DSMB review. Also, study activities could be suspended if the manufacturer of any constituent study agent requires stoppage of agent use for safety reasons (e.g., product recall). If any death occurs in the study and is assessed to be related to the study treatment device by the study site investigator, enrollment in the trial will be halted, pending DSMB review. A manuscript is to be published to share our work with the broader diabetes community globally.

## 3. Results and Progress

In August 2022, the Uganda study team was trained for five weeks on the protocol, CGM use, interpretation of CGM and SMBG data, and research principles by the Minnesota study team. Video calls every 2–4 weeks and in-person visits by the Minnesota team twice per year provide ongoing monitoring and mentorship. Recruitment began in August 2022. Two of the planned four cohorts have completed the study and the third cohort is in progress. As of August 2025, 136 participants had been enrolled, ~75.0% of the target sample size of 180 in Figure S1 [19]. We hope to complete the enrolment of participants by 31 March 2026 and complete the last patient follow up by 30 April 2027. The study will be completed in August 2027 with a manuscript preparation.

## 4. Discussion

This protocol describes the background, design, and organization of a randomized clinical trial to determine whether CGM wear improves glucose time-in-range (which could include reducing time in both hyper- and hypoglycemia) in children and youths with type 1 diabetes in Uganda. Importantly, it involves a cost-effectiveness analysis since the economics of CGM use in this region may be quite different from what has been found in the high-income countries where it has become standard-of-care. The study involves a large number and wide age range of children and young adults from a low-resource country, making the results potentially generalizable for wider applicability in sub-Saharan Africa. Involvement of the local Ministry of Health economists allows assessment of the real-world practicality of this intervention in improving diabetes care in a low-resource setting.

CGM has rapidly become standard-of-care in the US and Europe but is virtually unknown in resource-poor settings. A decade ago the most frequent cause of TID-related death in Africa was reported to be lack of insulin [20]. While insulin and diabetes test strips are still far from universally available worldwide, this is no longer the case for most children in Uganda and in much of the world where CDiC and Life for a Child have stepped in to provide basic diabetes therapies. When children were dying because insulin and test strips were not at all available, the goal was to provide a somewhat arbitrarily determined “minimal level of acceptable care”, which was primarily intended to keep them alive. However, marked improvements in insulin and test strip availability now challenge the assumption of what constitutes “acceptable care”, and raise questions about the quality of care. This is because diabetes metabolic control is still poor in these children, and they are regularly experiencing catastrophically high and low blood glucose levels [21,22,23,24,25].

All patients in this study, including those in the control group, will receive more intensive education, greater attention from the diabetes team, and more test strips than are commonly available today. If this approach results in similar levels of improvement in glucose TIR in control subjects using SMBG compared to patients who use unblinded CGM, this study will have performed an important service by demonstrating that there is no need for this expensive therapy and that, instead, more focus needs to be placed on patient education and interaction with the medical team. But if CGM leads to significantly greater improvement in diabetes metabolic control by reducing hyper- and hypoglycemia, then the ethical question is not whether to provide this therapy in resource poor settings but how to make it affordable. This is similar to the issue that arose when HIV/AIDS therapies first became available. Such decisions must be guided by data obtained in the specific and unique settings found in low, low-/middle income nations. The goal of this protocol is to obtain these data.

## Figures and Tables

**Table 1:** Overview of patient schedule activities.

Time (−1 Week, then Monthly)	−1 wk	0	1	2	3	4	5	6	7	8	9	10	11	12
Visit Number	1	2	3	4	5	6	7	8	9	10	11	12	13	14
**Informed Consent/Assent**	X													
**Initial Eligibility Screening, including wearing a single blinded Libre Pro CGM for at least 7 days**	X													
**Final Eligibility, Randomization**		X												
**Complete Medical History**	X													
**Interim Medical History**		X	X	X	X	X	X	X	X	X	X	X	X	X
**Complete Physical Exam**	X													
Limited/Directed Physical Exam *		X	X	X	X	X	X	X	X	X	X	X	X	X
**Physical Activity History**		X	X	X	X	X	X	X	X	X	X	X	X	X
**Pregnancy inquiry (as appropriate)**	X	X	X	X	X	X	X	X	X	X	X	X	X	X
**Prior/Concomitant Meds**	X	X	X	X	X	X	X	X	X	X	X	X	X	X
**Adverse Event Assessments**	X	X	X	X	X	X	X	X	X	X	X	X	X	X
**Daily Insulin U/kg/day, Type of Insulin**	X	X	X	X	X	X	X	X	X	X	X	X	X	X
**Severe Hypoglycemia History**	X	X	X	X	X	X	X	X	X	X	X	X	X	X
**Clinic POC HbA1c**	X				X			X			X			X
Control Group Months 0–6: Libre Pro (blinded) CGM placed in clinic and returned to clinic the following month, test strips dispensed in clinic and SMBG downloaded monthly **		X	X	X	X	X	X							
**CGM Group Months 0–12 and Control Group Months 7–12: Libre 2 (unblinded) CGMs dispensed in clinic monthly, worn continuously, and completed CGMs returned monthly to clinic**		X	X	X	X	X	X	X	X	X	X	X	X	
**Insulin Dispensed**		X	X	X	X	X	X	X	X	X	X	X	X	X
Validated Questionnaires ***	X							X						X
**Patient Education**	X	X	X	X	X	X	X	X	X	X	X	X	X	X

* monthly physical exam, ** done monthly for 6 months, *** Questionnaire filled every 6 months.

## Data Availability

Data from this trial will be made available at completion of the study, upon reasonable request to the authors.

## Supplementary Materials

The following supporting information can be downloaded at: https://www.mdpi.com/article/10.3390/mps9020043/s1, Figure S1: CONSORT 2025 Flow Diagram; Table S1: SPIRIT 2025 checklist of items to address in a randomized trial protocol*. References [19,26] are cited in the Supplementary Materials.

## Abbreviations

CGM	Continuous glucose monitoring
HbA1c	Hemoglobin A1c
IDF	International Diabetes Federation
POC	Point of care
RCT	Randomized, controlled trial
SMBG	Self-monitoring of blood glucose (meter, test strips)
T1D	Type 1 diabetes
TIR	Time-in-range, glucose 70–180 mg/dL (3.9–10.0 mmol/L)
UADE	Unanticipated Adverse Device Effect

## Appendix A

**Table A1:** Protocol development: challenges identified and the approaches to resolving them.

Challenges Recognized During Protocol Planning	Planned Approach
When determining overall study goals and plans, ensuring local significance and impact requires a “ground up” rather than a “top down” approach. Local community support is important.	Dr. Moran has been visiting Uganda at least annually since 2007, as part of a clinical collaboration between Mulago Hospital and the University of Minnesota. The strong personal ties that have developed over the years between her and Mulago pediatric endocrinologist Dr. Piloya-Were helped foster collaboration in the development of this protocol and the NIH grant. The project originated from concerns raised by Dr. Piloya-Were about her patients and their needs. She and Dr. Moran worked together on all aspects of this protocol. They sought input from their colleagues and from a local diabetes patient and family association (the Sugar Cubes) during the planning. Dr. Moran’s primary role was to organize the project goals into standard research protocol and grant application formats.
Identifying participating sites for a multicenter study should consider subject availability, the experience of the site investigators, and the potential for productive investigator collaboration.	Mulago Hospital, where Dr. Piloya-Were practices, does not have sufficient patients to complete the entire study. She chose to collaborate with Drs. Catherine Nyangabyaki and Silver Bahendeka at St. Francis Hospital, Nsambya, located about 5 km from Mulago. Mulago and Nsambya are the two largest pediatric diabetes clinics in the country. Drs. Piloya-Were, Nyangabyaki, Bahendeka and Moran have all previously worked successfully together.
Initial discussions focused on determining the best and most doable intervention to improve diabetes metabolic control.	In order to move closer towards diabetes advancements that are standard for patients in well-resourced countries, we considered CGM technology, analog insulin use, or a combination of the two in a 2 × 2 study design. It was decided that doing both was too much of a clinical change all at once for the local teams and patients, and too complex a research protocol for a team that had no experience with RCTs. Of the two, the Ugandan team felt that CGM was the more important first step.
No previous CGM study had been done under these conditions, so there were no relevant data available for power analysis.	To address this, we performed a pilot and feasibility study with a single blinded sensor wear in 78 East African youths with TID to determine patient level of interest, and to assess potential problems in this humid, equatorial environment (e.g., equipment malfunction, difficulty with site adhesion, skin irritation or infection) [3]. The pilot study provided critical data for sample size calculation.
Global research generally requires at least some degree of local institutional experience in grant submission and grant management.	Global Health Uganda, a grants management organization, has extensive experience and also has a longstanding formal relationship managing grants with the University of Minnesota (UMN). Thus, this was a logical partnership. We originally tried to add a site in another East African country, but despite the best efforts of their team and ours, they did not have sufficient research administrative support to proceed.
Global research involves addressing a myriad of local logistical and regular requirements and getting appropriate approvals.	Prior to grant submission, Dr. Moran and Research Manager Ms. Pappenfus spent time in Uganda with Dr. Piloya-Were obtaining input and permission from the Ministry of Health and the Ugandan Drug Development Authority. They partnered with Ministry of Health economists in developing the cost-effectiveness component of the protocol. Importing the CGM systems into the country was also a concern. Changing Diabetes in Children (CDiC, Novo Nordisk) provides free insulin, test strips, and HbA1c point-of-care machines for Ugandan youths with diabetes. We arranged to leverage their supply infrastructure by providing some salary support for their Ugandan procurement officer, to ensure the ability to follow all legal requirements and permits to ship CGM supplies into Uganda.
Research concerns, Ugandan team: Ugandan team members have experience with observational research, but not RCTs.	An initial intensive research education program in Uganda was performed at study start up with ongoing education continuing throughout the study. Monitoring is done in the spirit of education. In addition to continuous remote monitoring, the UMN team visits Uganda twice per year for monitoring and, as needed, research re-education. Some of the Ugandan team have visited Minnesota and we plan to bring each of them (physicians, nurses, dietitians) to Minnesota over the grant period for more intensive training in research methods.
Research concerns, participants: Recruitment and language barriers.	Recruitment is often the biggest hurdle in clinical studies. Our pilot study demonstrated that Ugandans with T1D were eager to participate in a CGM study. Language can also be a hurdle in international studies. English is the official language of Uganda, spoken by all school students and most adults. The Ugandan staff are all bilingual in English and the local language consent forms are available in both languages, and education is delivered in both languages.
Clinical concerns, Ugandan team: Drs. Piloya-Were and Nyangabyaki have pediatric diabetes training, but the nurses and dietitians on their teams have had little formal diabetes training and frequently are required to rotate to different clinical services. Because only minimal glucose data are commonly available, the Ugandan team does not have much experience with pattern recognition. CGM technology is not available in Uganda.	The budget allows salary support to train and retain three nurses, one dietitian, and two junior physicians (medical officers), who will remain with the study for its entirety, ensuring continuity. The pilot study familiarized the Ugandan team with CGM technology, and the ongoing advanced education on how to use this technology was built into the study protocol. Both initial and on-going staff education is planned, reviewing all aspects of T1D and its management, delivered in person and virtually. In addition, the Minnesota and Uganda teams together review patterns on individual participant CGM downloads monthly in a virtual format. Subspecialty training of nurses and dietitians is not common in Uganda, and thus this project is increasing local capacity for experienced pediatric diabetes care.
Clinical concerns, participants: T1D patients in Uganda are not accustomed to adjusting their own insulin doses. There is shame and stigma associated with diabetes and the fear that people with T1D will never lead “normal” lives.	The patients, who have never before been exposed to extensive glucose data, have minimal understanding of insulin action and its relation to food, activity, or hypoglycemia. An extensive patient education program is planned with didactic group education at each visit, and individual pattern recognition using their own (SMBG or CGM) data. There is also concern about the social stigma of having diabetes. CGM patches will be provided that are appropriate to their skin color so as to be less conspicuous. We are working with an NGO called the Sonia Nabeta Foundation that is empowering young adults with T1D to be peer leaders. Many of the Minnesota team members have diabetes themselves and serve as role models to show that people with diabetes can lead normal lives, including holding meaningful jobs and having a family.
Clinical concerns, Minnesota team: The younger members of the team have no experience with human (regular and NPH) insulin.	Older members of the Minnesota team and those who have previously worked in low-resource countries are experienced with these insulins and will teach the others.
If our hypothesis is correct and CGM improves diabetes metabolic control in this setting, it is meaningless unless this therapy can actually be introduced to patients there.	For the results of a positive study to impact patient care in Uganda and other sub-Saharan African countries, CGM needs to be cost effective in these low-resource settings. Thus, we involved health economists from the Ministry of Health from the beginning in project planning, and they will have an important role analyzing the data at study end.
