# Supplementary material for: A Study Protocol for a Randomized, Controlled Trial: Improving Glucose Time-in-Range in Diabetes in African Youth (DAYTime)
Source: Methods Protoc. 2026 Mar 8;9(2):43. doi: 10.3390/mps9020043 (PMC13010732; doi:10.3390/mps9020043)
Supplement: Supplementary file 1 [file mps-09-00043-s001.zip › CONSORT_2025_flow_diagram - Filled 9-jan2026.pdf]

Figure S1: CONSORT 2025 Flow Diagram

Flow diagram of the progress through the phases of a randomised trial of two groups (that is, enrolment, intervention allocation, follow-up, and data analysis)

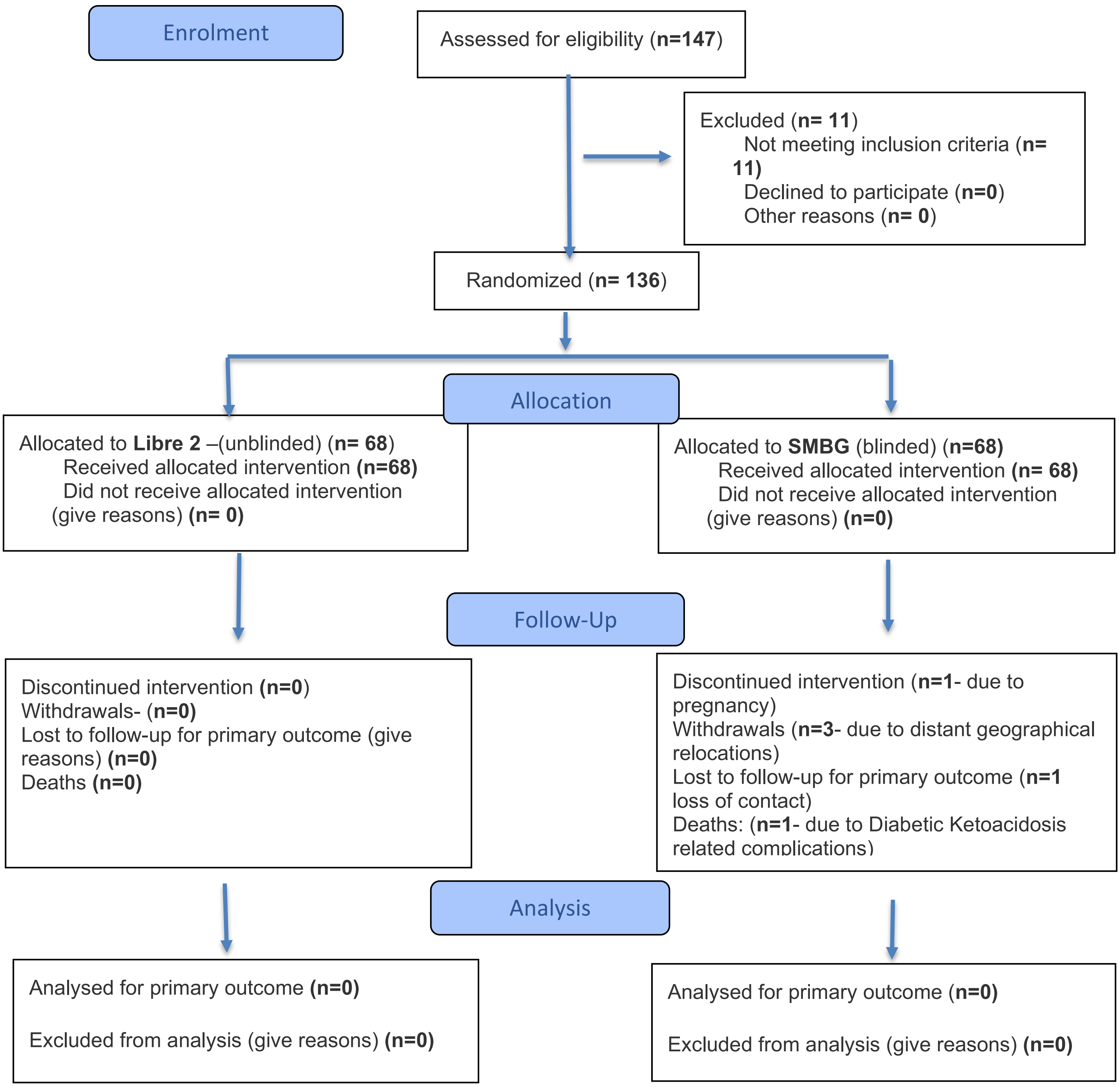

Citation: Hopewell S, Chan AW, Collins GS, Hróbjartsson A, Moher D, Schulz KF, et al. CONSORT 2025 Statement: updated guideline for reporting randomised trials. BMJ. 2025; 388:e081123. https://dx.doi.org/10.1136/bmj-2024-081123
© 2025 Hopewell et al. This is an Open Access article distributed under the terms of the Creative Commons Attribution License (https://creativecommons.org/licenses/by/4.0/), which permits unrestricted use, distribution, and reproduction in any medium, provided the original work is properly cited.
